# Self-medication practice among the general public in Jordan: a cross-sectional study

**DOI:** 10.3389/fpubh.2024.1433464

**Published:** 2024-10-17

**Authors:** Sawsan M. A. Abuhamdah, Abdallah Y. Naser

**Affiliations:** ^1^Department of Biopharmaceutics and Clinical Pharmacy, School of Pharmacy, The University of Jordan, Amman, Jordan; ^2^Department of Pharmaceutical Sciences, College of Pharmacy, Al Ain University, Abu Dhabi, United Arab Emirates; ^3^AAU Health and Biomedical Research Center, Al Ain University, Abu Dhabi, United Arab Emirates; ^4^Department of Applied Pharmaceutical Sciences and Clinical Pharmacy, Faculty of Pharmacy, Isra University, Amman, Jordan

**Keywords:** general public, Jordan, over-the-counter, public, self-medication

## Abstract

**Objectives:**

The phenomenon of self-medication is a noteworthy public health concern that is increasingly prevalent on a global level, particularly in developing nations. This study aims to explore the prevalence of self-medication of over-the-counter (OTC) medications among the general public in Jordan.

**Materials and methods:**

This cross-sectional online survey study was conducted in Jordan between November and December 2022. An online questionnaire link was distributed to the study participants through social media platforms (Facebook, WhatsApp, and Instagram). The questionnaire tool for this study was adapted from a previously developed questionnaire by Tesfamariam et al. The questionnaire tool comprised four sections that examined participants’ demographic characteristics, knowledge of OTC self-medication, attitudes toward OTC self-medication, and associated practices. Binary logistic regression analysis was used to identify predictors of OTC self-medication practice.

**Results:**

A total of 1,218 individuals participated in this study. More than half of the study participants (56.9%) confirmed that they were currently using or had in the past year used medication(s) not prescribed by a healthcare specialist. Participants’ agreement level on statements that reflected a positive attitude toward self-medication practice ranged between 13.1 and 33.0%. The statement most commonly agreed with was that ‘OTC medications are safe but I would seek a physician’s advice before using them’ (57.7%). Individuals aged 36–40 years and 51 years and over, those who were married or divorced, those who were employed, and those who had chronic diseases were more likely to practice self-medication compared to others (*p* < 0.05).

**Conclusion:**

The current study findings suggest a significant lack of awareness among the general population in Jordan regarding the expected efficacy of OTC medications. A restricted level of agreement was observed among the participants concerning the behavior of self-medication.

## Introduction

Self-care is the most substantial public health resource in the healthcare system, according to the World Health Organization (WHO). It encompasses first aid in daily situations, non-drug self-treatment, social support during sickness, and self-medication (1). Self-medication means using medications to treat disorders or symptoms that individuals have independently diagnosed themselves or the occasional or regular use of prescribed drugs for managing recurring or chronic conditions or symptoms (1). This practice also extends to medication administration to family members, particularly when caring for the older adult or children (1). Self-medication medicines refer to medications individuals can purchase without a prescription from a healthcare professional, also known as over-the-counter (OTC) or non-prescription drugs (2).

The self-medication practice is a significant public health issue on a global scale, showing a prevalence ranging from 11.7 to 92% in various regions of the world (3, 4). Globally, self-medication rates are rising, especially in developing countries. Several factors lead to self-medication, including difficult access to professional healthcare services, lack of awareness, presence of medications outside pharmacies, economic constraints, the desire for self-care, extensive advertising, compassion toward sick family members, and misbeliefs (5).

Some traditional medicines can be used without a prescription from a physician. Nevertheless, the majority of medications, particularly antibiotics, require stringent control and regulation for their use (6). The appropriate practice of self-medication can offer advantages to people and healthcare systems. These benefits encompass lowering healthcare expenses, decreasing work absenteeism, alleviating the burden on healthcare professionals, saving limited medical resources for more critical conditions, and lessening the time spent waiting for medical appointments (7). On the other hand, inappropriate usage of self-medication can lead to undesired consequences. Studies have highlighted potential risks associated with self-medication, including incorrect diagnosis, adverse drug reactions arising from inappropriate pharmaceutical usage, improper length of use, and excessive dosing (8, 9). Improper self-medication can result in treatment delays, an escalation in polypharmacy, wastage of resources, interactions with commonly used medications, and irrational use of medicines (10). Furthermore, excessive antibiotic use contributes to the universal spread and emergence of drug-resistant pathogens (11).

Previous research studies in Jordan have significantly contributed to our understanding of self-medication patterns among Jordanians (12). However, an unexplored research gap exists concerning self-medication practices of OTC medications within the general Jordanian population. Therefore, this study aims to explore the prevalence of self-medication of OTC medications among the general public in Jordan.

## Materials and methods

### Study design

This cross-sectional online survey study was conducted in Jordan between November and December 2022.

### Study population

This study explored its study objectives among the general public in Jordan. The study inclusion criteria were adult males or females aged 18 years and older who currently live in Jordan. No restrictions were placed on the participants’ socioeconomic status or their health insurance plans. Participants who did not provide their consent for participation were excluded from the study.

### Participants recruitment

This study employed a convenience sampling technique to recruit participants. An online questionnaire link was distributed to potential participants through social media platforms (Facebook, WhatsApp, and Instagram) to reach a wide range of participants from different socioeconomic groups. This enabled the researchers to collect data faster at lower costs. In countries like Jordan, the percentage of the population active on social media platforms is ever-increasing, and participant recruitment was therefore facilitated. The study inclusion criteria were mentioned in the cover letter accompanying the questionnaire link. Participants were asked to participate in the study if they met the inclusion criteria for the study.

### Questionnaire tool

The questionnaire tool for this study was adapted from a previously developed questionnaire by Tesfamariam et al. (13). The questionnaire tool comprised four sections that examined participants’ demographic characteristics (six multiple-choice questions), knowledge of OTC self-medication, attitudes toward OTC self-medication, and their associated practices. The assessment of knowledge was conducted through the administration of a questionnaire consisting of 10 items (five yes/no format questions and five multiple-choice questions). These items were specifically developed to evaluate the respondents’ overall understanding of OTC medications, including their awareness of indications, contraindications, adverse effects, and proper usage of such treatments. The measurement of attitude consisted of 11 items and used a five-point Likert scale that ranged from strongly disagree to strongly agree. This section assessed perceptions regarding the safety, efficacy, accessibility, and usage of OTC medications. Finally, the evaluation of practice entailed the administration of a set of six questions (multiple-choice format) aimed at assessing healthcare professionals consulted before practicing OTC self-medication, reasons for OTC self-medication, medications categories used for OTC self-medication, whether participants had ever taken more than the recommended dose of OTC medications, and whether they had ever experienced adverse effect due to OTC medications.

### Face and content validity

The face and content validity of the original questionnaire were assessed by convening a panel of experts from various disciplines, including pharmacy, epidemiology, pharmacoepidemiology, public health, and environmental health, who engaged in a comprehensive debate. The interviewers consisted of fifth-year pharmacy students who had received training to ensure the clarity of the items to maximize both within-group and between-group consistency among the raters. The internal consistency (reliability) of the attitude scale was deemed acceptable using Cronbach’s alpha of *α* = 0.723.

### Questionnaire translation

To facilitate the recruitment of study participants in Jordan, we translated the questionnaire tool into Arabic using the forward-backward translation technique. In so doing, we focused on the meaning of the translated items, not on word-by-word translation.

### Pilot phase

A pilot study using the Arabic version of the questionnaire was then conducted with 30 participants in Jordan who met the inclusion criteria. The participants were requested to comment on clarity and comprehensibility regarding the questionnaire and whether any of the questions were not clear. Finally, they were asked if any of the questions seemed inappropriate or offensive. The participants confirmed that all questions were clear and easy to understand.

### Sample size

The sample size for this study followed the procedure adopted in Tesfamariam et al.’s original study (13). The minimum required sample size for this study was estimated using the following formula: *n* = *Z*^2^*P (1−*P*)/*d*^2^. The total sample size (*n*) was determined based on the following assumptions: A proportion (*P*) of 0.5 was used due to the absence of prior similar research, a *Z* statistic of 1.96 was used to achieve a 95% level of confidence, a degree of precision (*d*) of 0.05 was selected, and a 5% non-response rate was accounted for. The sample size was adjusted by considering the design effect (1.5), given that a two-stage cluster sampling method was employed as the sampling design in the original study. Consequently, the final sample consisted of 609 participants.

### Statistical analysis

The Statistical Package for Social Science software version 29 was used to analyse the data for this study. Categorical data were presented as frequencies and percentages. Binary logistic regression analysis was used to identify predictors of OTC self-medication practice. Participants’ sociodemographic characteristics formed the independent variables in the regression model (covariates). The dependent variable used in the regression variable was the use of medication(s) not prescribed by a healthcare specialist, which was confirmed by asking the participants (‘Are you currently using or have you used medications not prescribed by a healthcare specialist in the previous year?’). Cronbach’s alpha was used to examine the reliability of the attitude scale. The odds ratio was presented with its corresponding 95% confidence interval. The significance level was assigned as a *p*-value below 0.05.

## Results

### Participants’ demographic characteristics

Table 1 presents the demographic characteristics of the study participants. A total of 1,218 individuals participated in this study. More than half (55.9%) were females, almost one-third (36.5%) were aged 18–23 years, and around half (50.7%) were single. The monthly income category for one-third of the study participants (32.0%) was less than JOD 500. Fewer than half (44.9%) of the study participants reported that they were employed. Around 28.1% reported that they had a chronic disease history. More than half of the study participants (56.9%) confirmed that they were currently using or had in the past year used medication(s) not prescribed by a healthcare specialist.

**Table 1:** Participants’ demographic characteristics.

Variable	Frequency	Percentage
Gender		
Females	681	55.9%
Age categories		
18–23 years	445	36.5%
24–30 years	245	20.1%
31–35 years	156	12.8%
36–40 years	128	10.5%
41–45 years	108	8.9%
46–50 years	78	6.4%
51 years and over	58	4.8%
Marital status		
Single	618	50.7%
Married	491	40.3%
Divorced	72	5.9%
Widowed	37	3.0%
Monthly income category		
Less than 500 JOD	390	32.0%
500–1,000 JOD	500	41.1%
1,001–1,500 JOD	241	19.8%
More than 1,500 JOD	87	7.1%
Employment status		
Retired	53	4.4%
Unemployed	259	21.3%
Employed	546	44.9%
University student	360	29.4%
Do you have any chronic disease history?		
Yes	341	28.0%
No	877	72.0%
“Are you currently using or have you used medications not prescribed by a health care specialist in the previous year?”		
Yes	691	56.7%
No	527	43.3%

### Knowledge related to self-medication

Table 2 presents participants’ responses to knowledge items. Nearly half (48.2%) of the study participants confirmed that ‘medicines are always used on the prescription of a doctor’. More than half (63.1%) identified that not all OTC medications are safe and effective. Only 41.0% identified that OTC medications are not approved for self-care. More than half (63.5%) identified that ‘not all OTC medications are safe when taken along with prescribed drugs’. The majority (74.7%) identified that not all OTC medications could be used after their expiry date.

**Table 2:** Knowledge related to self-medication.

Variable	Frequency	Percentage
Medicines are always used on the prescription of a doctor? (Yes)	587	48.2%
All over the counter drugs are safe and effective? (No)	769	63.1%
Over the counter drugs are approved for self-care? (No)	499	41.0%
All over the counter drugs when taken along with prescribed drug are safe? (No)	774	63.5%
Over the counter drugs could be used after their expiry date? (No)	910	74.7%
Over the counter drugs are used for treating diseases like		
Hereditary diseases	94	7.7%
Minor illnesses and injuries	694	57.0%
Do not know	430	35.2%
Which of the following drugs fall under OTC drugs? (multiple answers question)		
Analgesics	794	65.2%
Anti-cold	736	60.4%
Antipyretics	648	53.2%
Anti-microbials	265	21.8%
Over the counter drugs can		
Sometimes cause side-effect(s)	562	46.1%
Mostly cause side-effect(s)	291	23.9%
Never cause side-effect(s)	134	11.0%
Do not know	231	19.0%
While using Over the Counter drugs, caution should be taken mostly in: (multiple answers question)		
Pregnancy	922	75.7%
Lactation	754	61.9%
Older adult	732	60.1%
Children	643	52.8%
Adolescent/middle adults	296	24.3%
If suspected side-effect(s) are seen, then one should: (multiple answers question)		
Immediately stop using the drug	730	59.9%
Take low dose until side effect(s) subside	262	21.5%
Continue taking the drug regardless the side effect(s)	182	14.9%
Report to a Doctor or Pharmacist	707	58.0%

More than half of the study participants (57.0%) reported that OTC medications can be used to treat minor illnesses and injuries. Around one-fifth (21.8%) incorrectly classified anti-microbials as OTC medications. Around 11.0% incorrectly believed that OTC medications could never cause side effects. The most commonly reported patient population for whom caution should be taken while using OTC medications was pregnant women (75.7%). Nearly two-thirds (59.9%) of the study participants reported that they would stop taking OTC medications immediately if suspected side effects occurred.

### Attitudes toward self-medication practice

Table 3 presents participants’ attitudes toward self-medication practice. Participants’ agreement level on statements that reflected a positive attitude toward self-medication practice ranged between 13.1 and 33.0%. The statement most commonly agreed with was that ‘OTC medications are safe but I would seek a physician’s advice before using them’ (57.7%). The statement least commonly agreed with was that ‘OTC medications are not affected by storage conditions like temperature, moisture and direct sunlight’ (19.9%).

**Table 3:** Attitudes toward self-medication practice.

Attitude statement	Strongly disagree	Disagree	Neutral	Agree	Strongly agree
Over the counter drugs that are used for self-medication are safe	11.7%	20.7%	34.7%	23.6%	9.4%
Over the counter drugs are cheaper and convenient	9.4%	28.1%	31.9%	23.6%	7.0%
Paracetamol in overdose is a powerful poison	5.5%	10.6%	33.1%	35.8%	15.0%
Over the counter drugs can modify or alter the action of another drug	6.6%	17.7%	31.7%	33.2%	10.8%
All over the counter drugs can be used in case of pregnancy	26.0%	26.6%	25.9%	16.0%	5.5%
Pain killers when taken on an empty stomach does not cause gastritisa	17.9%	25.8%	26.9%	21.7%	7.7%
Over the counter drugs are not affected by storage conditions, like temperature, moisture and direct sunlight	29.3%	25.5%	25.2%	14.2%	5.8%
Liquid medicines could be used when opened after 1 month	14.5%	23.3%	30.5%	26.9%	4.8%
Eye/Ear drops could be used after 1 month of opening	18.8%	21.4%	29.3%	24.1%	6.4%
It is better I do not take over the counter drug when I am ill	6.9%	23.5%	33.6%	27.1%	8.9%
Over the Counter drugs are safe but would seek a physician advice before using it	3.5%	9.6%	29.1%	40.9%	16.9%

### Self-medication practices pattern

Table 4 presents participants’ self-medication practice patterns. The person the participants most frequently consulted before using OTC medications was their pharmacist (33.7%). Nearly two-fifths (38.9%) of the study participants reported that they consumed OTC medications when their symptoms were minor. The most commonly reported reason for using OTC medications was their lower cost. Analgesics were the most commonly reported (14.7%) class of medications used for self-medication. Nearly three-quarters (72.4%) of the participants reported that they had taken more than the recommended dose of their OTC medications. Over half (55.6%) reported that they had experienced side effects due to the use of OTC medications.

**Table 4:** Self-medication practices pattern.

Variable	Frequency	Percentage
With whom did you consult before using over the counter drugs? (*n* = 691) (multiple answers question)		
Pharmacist	233	33.7%
Doctor	229	33.1%
Friends/relatives	167	24.2%
Leaflet	83	12.0%
Internet and mobile applications	91	13.2%
When did you consume over the counter drugs? (*n* = 691)		
When symptoms are minor/manageable	269	38.9%
Whenever I feel sick	260	37.6%
When I cannot visit doctor	162	23.5%
Common reason(s) for using over the counter drugs is: (multiple answers question)		
Low cost	129	18.6%
Time saving	101	14.6%
Easy accessibility	97	14.1%
Safe and well tolerable	72	10.4%
Which categories of medications are mostly preferred by you for self-medication? (*n* = 691)		
Analgesics	102	14.7%
Cough and cold preparation	68	9.8%
Antipyretic	65	9.4%
Vitamin	57	8.3%
Anti-inflammatory	27	3.9%
Anti-allergic	26	3.8%
Antacids	26	3.7%
Anti-diarrheal	24	3.5%
Have you ever taken Over the Counter drug(s) more than the recommended dose? (*n* = 691)		
Yes	500	72.4%
No	191	27.6%
Have you ever experienced adverse effect from Over the Counter drugs? (*n* = 691)		
Yes	384	55.6%
No	307	44.4%

### Factors influencing self-medication

Binary logistic regression analysis identified that individuals aged 36–40 years and 51 years and over, those who were married or divorced, those who were employed, and those who had chronic diseases were more likely to practice self-medication compared to others (*p* < 0.05); see Table 5.

**Table 5:** Predictors of self-medication practice among the general public.

Variable	Odds ratio of self-medication practice	95% confidence interval
Gender		
Females (reference category)	1.00	
Males	1.14 (0.91–1.44)	0.256
Age groups		
18–23 years (reference category)	1.00	
24–30 years	1.18 (0.87–1.62)	0.292
31–35 years	1.13 (0.78–1.63)	0.521
36–40 years	1.53 (1.02–2.29)	0.039*
41–45 years	1.54 (1.00–2.37)	0.052
46–50 years	1.64 (1.00–2.70)	0.052
51 years and over	1.88 (1.06–3.36)	0.032*
Marital status		
Single (reference category)	1.00	
Married	1.44 (1.13–1.83)	0.003**
Divorced	1.85 (1.11–3.10)	0.019*
Widowed	1.93 (0.95–3.91)	0.069
Employment status		
Retired (reference category)	1.00	
Unemployed	1.18 (0.90–1.54)	0.226
Employed	1.69 (1.22–2.36)	0.002**
University student	1.23 (0.77–1.97)	0.385
Monthly income for the family		
Less than 500 JD (reference category)	1.00	
500–1,000 JD	0.60 (0.33–1.11)	0.101
1,000–1,500 JD	0.87 (0.49–1.57)	0.652
1,500 JD and above	0.64 (0.35–1.16)	0.138
Chronic disease history		
No (reference category)	1.00	
Yes	1.89 (1.45–2.46)	<0.001

*p < 0.05; **p < 0.01.

## Discussion

The results of this cross-sectional study highlight some trends in self-medication practices among the general public in Jordan: Individuals aged 36–40 years and 51 years and over, those who were married or divorced, those who were employed, and those who had chronic diseases were more likely to practice self-medication compared to others.

Notably, in our study, more than half of the participants (63.1%) acknowledged that not all OTC medications are safe and effective. Concerning the safety of OTC medications, this result indicates that most of the general public in Jordan has a reasonable understanding of the potential risks associated with OTC self-medication practices. Few studies have demonstrated that consumers understand the importance of using these medications carefully (14) and acknowledge their associated possible adverse effects (15). This heightened awareness is crucial because some consumers perceive OTC medications as entirely harmless, leading them to underestimate the associated risks (16).

However, concerning the efficacy of OTC medications, this finding is worrisome as it underscores a significant lack of awareness among the general population in Jordan regarding the intended efficacy of OTC medications. The practice of self-medicating with OTC medications is considered both efficacious and secure. Nevertheless, improper usage due to a lack of awareness about their potential adverse effects and drug interactions can lead to severe outcomes, particularly for vulnerable groups such as breastfeeding mothers, pregnant women, the older adult, and children (17).

Our study revealed that only 41.0% of the respondents identified that these medications are not approved for self-care. This finding is worrying as it shows that the general public has a severe deficit of understanding regarding the proper use of OTC medications. It implies that a significant percentage of people do not fully understand that OTC medications are intended for self-administration without the help of a healthcare provider (18). In comparison with previous studies that have explored public knowledge of OTC medication, research conducted in Saudi Arabia reported poor knowledge about OTC medications among 69.7% of the participants (19). In contrast, a study conducted in Afghanistan demonstrated that 65.2% of the participants had good knowledge regarding the usage of OTC medications (20). Similarly, another study conducted in Jordan found that the majority of participants reported good knowledge about OTC medications (21). The variety in understanding OTC self-medication can be explained by variations in health-related socio-cultural knowledge, behaviors, attitudes, and beliefs among the general public.

Medications used while pregnant necessitate a thorough evaluation of the advantages to the mother and the potential risks to the developing fetus. Due to the diverse pharmacokinetic and physiological alterations that arise during pregnancy, choosing appropriate drugs for pregnant women poses challenges for healthcare professionals (10, 22). One of the significant findings from this cross-sectional study is that the most commonly reported patient population for whom caution should be taken while using OTC medications was pregnant women (75.7%). This result highlights the general public’s awareness of the potential risks associated with self-medication during pregnancy, which is a critical factor of responsible healthcare management. The high level of concern expressed by the study participants regarding the use of OTC medications during pregnancy is consistent with previous research. A study conducted among pharmacy and medical students in Ethiopia reported that approximately 91.3% of participants agreed that caution should be exercised primarily when using OTC medications during pregnancy (23). Similarly, another study among university and college students in Brunei Darussalam found that about half (50.1%) of the participants agreed that OTC medication could be used with caution during pregnancy and breastfeeding unless a warning label advised against their use (24). Additionally, comparable to the prevalence of self-medication reported worldwide among pregnant women, recent research in Jordan revealed that the practice of self-medication was not common, with only approximately one-third (33%) of pregnant women reporting practicing self-medication (12). The relatively lower prevalence might be attributed to pregnant women’s heightened awareness of the potential risks, particularly teratogenic effects, associated with using medicinal products during pregnancy (25).

Another important finding from this study is that a significant proportion of participants (33.7%) reported their pharmacist as the person they consulted before using OTC medications. This result highlights the essential role of pharmacists in promoting safe and responsible OTC self-medication practices among the general public. The WHO has also described the essential role of the pharmacist in self-medication (1). By comprehending the patient’s disease and providing pharmaceutical information, pharmacists can assist the general public in using self-medication safely (26). Consistent with our finding, a previous study conducted in Jordan observed that 58.8% of the participants sought information about self-medication by consulting a pharmacist in the pharmacy (27). Similarly, the pharmacist was one of the primary sources of drug self-medication information named by university students in a Jordanian survey (28). In addition, previous research found that more than 90% of participants in Western India preferred pharmacies as their basis for self-medication information (4). Studies conducted in Gorgan, North of Iran (29), Soba, Sudan (30), Minia, Egypt (31), and Coastal South India (32, 33) have demonstrated comparable outcomes.

Our study found that the most commonly reported reason for using OTC medications was their lower cost. This emphasizes the significant influence of affordability in shaping self-medication behaviors in the general population. At the community level, the WHO acknowledges that proper self-medication practices can effectively reduce healthcare expenses (1). Various studies have identified diverse causes for self-medication with OTC drugs. These reasons encompass cost-effectiveness, easy accessibility, time savings, and non-seriousness of the disease (13, 34–38). The finding that OTC drugs are preferred because they are more affordable aligns with previous research conducted in various countries. In a study conducted in Jordan, approximately 71% of the participants perceived self-medication as a more cost-effective option compared to seeking healthcare professional advice (27). A previous study from India revealed that a notable number of healthcare workers concurred that self-medication leads to savings on consultation fees (39). A study conducted in Riyadh, Saudi Arabia, found that over 40% of self-medicated patients cited the expense of doctor consultations as a significant factor leading to self-medication (40). Likewise, a separate study in Sudan revealed that half of the participants (50%) refrained from consulting with a doctor due to the financial burden involved (30). Based on these findings and considering the potential benefits of self-medication with OTC drugs when carried effectively, healthcare policymakers must emphasize responsible self-medication practices, as they can help alleviate acute pain, reduce physician interaction time, lower treatment costs (34), and lead to more cost-effective and efficient healthcare outcomes.

The largest OTC market in the world is for analgesics (41). Our finding showed that analgesics are the most commonly reported (14.7%) class of medications used for self-medication. Consistent with this finding, numerous other studies conducted in Jordan have revealed that analgesics are the most frequent self-medication drugs (21, 27). Moreover, in Jeddah and Makkah, Saudi Arabia, analgesics were found to be the most commonly used drugs among the general public, accounting for 65.6% of self-medication practices (42). Similarly, in the Western Region of Saudi Arabia, analgesics were the most frequently used medications, making up 44.0% of self-medication cases among the general population (43). In India, analgesics were identified as the most extensively used drugs for self-medication, accounting for 66.3% of cases (44). Other studies have reported similar patterns in which analgesics were commonly used for self-medication (45–50). However, analgesics can adversely affect consumers’ health. Distinctly, certain analgesics, like non-steroidal anti-inflammatory drugs (NSAIDs), have been linked to an increased risk of gastric ulceration and renal impairment (51). However, one-third of participants in a previous survey were unaware of the analgesic contraindications (52). Given that analgesics can negatively impact a consumer’s health, it is essential to increase public knowledge of the risks associated with their excessive use through health awareness programs.

Due to the potential for complications and adverse effects from medication misuse, self-medication can be risky (53). In our study, approximately one-third of participants (31.5%) reported having experienced side effects due to using OTC medications. The prevalence of side effects after using OTC medicines highlights the need for increased public awareness about potential risks and complications associated with these medications. Consistent with our findings, previous studies have also reported varying percentages of participants experiencing side effects from self-medication: 11.47% (54), 16.9% (55), 8.9% (56), and 19.2% (57). One of the prevalent negative consequences associated with self-medication practice is the inappropriate use of antibiotics, which can lead to the development of drug resistance (58, 59). Another group of medications often misused by self-medicated individuals is NSAIDs, which can result in severe side effects (53), as mentioned earlier. Moreover, the occurrence of misdiagnosis can also increase as a result of practices of irrational self-medication (9).

This study identified that the prevalence of self-medication among the study population has reached 56.9%. This cross-sectional study provides valuable insights into the demographic and health-related factors associated with self-medication practices among the general public in Jordan. The results indicated that individuals aged 36–40 years and 51 years and over, and those who were married or divorced, employed, and had chronic diseases, were more likely to practice self-medication compared to others (*p* < 0.05). Previous literature in the Mediterranean region demonstrated prevalence rates of self-medication between 35.4 and 83% (60). A study in the United Arab Emirates found a similar prevalence rate of self-medication (57.5%) among university students (61). A study in Nigeria reported a higher prevalence rate of self-medication (85.4%) and found that older age, having postsecondary education, and having good knowledge were predictors of practicing self-medication (62). A study in Thailand found a prevalence rate of self-medication of 88.2% (63). This study further reported that self-medication practice varied based on gender, marital status, level of education, occupation, underlying disease(s), drug allergy history, and health insurance (63). These findings can be attributed to variations in self-medication prevalence across age groups, which are influenced by multiple factors, including the frequency, severity, and type of disorders (64, 65). Notably, this practice is more common among individuals with chronic diseases and lower socioeconomic status (36).

Older adults might have acquired more knowledge about health and medications over time as they might have multiple comorbidities (therefore, using multiple medications, which is known as polypharmacy) and can easily access medications. Self-medication thus becomes prevalent in treating minor health problems that do not warrant professional consultation (66, 67). Individuals suffering from chronic diseases also tend to take medications quite often and are aware of the action of different drugs, which increases the chances of self-medication to manage symptoms that recur over time (68).

These findings shed light on the patterns of self-medication among various population groups and underscore the importance of targeted interventions to promote appropriate self-care behaviors. Moreover, a previous review indicates mixed findings regarding the associations between self-medication practices and the age, gender, and socioeconomic status of participants (69); some studies reported positive associations, while others reported negative associations. The relationship between these factors and self-medication prevalence appears to be complex and may vary across populations.

This is among the first studies that have examined self-medication practice among the general public in Jordan without restricting the study population to a specific study group or class of medications. The large sample size in this study increases the statistical power of our estimates and provides more accurate information related to self-medication practice among the general public in Jordan. This study has limitations, though. The cross-sectional study design restricted our ability to follow up and examine causality among the study variables. The use of the convenience sampling technique and the recruitment of study participants through social media may have affected the generalisability of our study findings due to the possibility of sampling bias. Also, desirability bias may occur if participants give responses they consider more socially acceptable rather than their true beliefs and behaviors. Recall bias could further result when participants inaccurately remember events in the past. Also, analysis based on essential sociodemographic characteristics such as rural vs. urban location, educational level, and socioeconomic condition is lacking. Therefore, our study findings should be interpreted carefully.

## Conclusion

The findings of this study have shown that a large proportion of the general public in Jordan lacks adequate knowledge about the efficacy of OTC medications and, at the same time, has inadequate consensus on the practice of self-medication. In addition, the results have shown that self-medication is significantly more common in the age groups between 36 and 40 years and above 51 years, among married or divorced people, those who work, and those with chronic diseases. These insights, therefore, suggest launching appropriate educational campaigns and healthcare interventions concerning safe self-medication, especially in these higher-risk groups, to ensure that potential health risks associated with the improper use of OTC medication can be averted.

## Data Availability

The original contributions presented in the study are included in the article/supplementary material, further inquiries can be directed to the corresponding author.
